# Sarcopenia prediction model based on machine learning and SHAP values for community-based older adults with cardiovascular disease in China

**DOI:** 10.3389/fpubh.2025.1527304

**Published:** 2025-05-21

**Authors:** Peil Yu, Xinxin Zhang, Guoxuan Sun, Ping Zeng, Chu Zheng, Ke Wang

**Affiliations:** ^1^Department of Biostatistics, School of Public Health, Xuzhou Medical University, Xuzhou, Jiangsu, China; ^2^Center for Medical Statistics and Data Analysis, Xuzhou Medical University, Xuzhou, Jiangsu, China; ^3^Jiangsu Engineering Research Center of Biological Data Mining and Healthcare Transformation, Xuzhou Medical University, Xuzhou, Jiangsu, China

**Keywords:** sarcopenia, CHARLS, cardiovascular disease, SHAP, diagnostic performance

## Abstract

**Background:**

Sarcopenia (SP), is recognized as a complication of cardiovascular disease (CVD), but few relevant diagnostic models have been developed. This study aims to establish an interpretable diagnostic model for the occurrence of SP in older adult CVD patients living in Chinese community-dwelling (CD).

**Methods:**

We randomly selected participants with CVD recruited from CHARLS from 2011 to 2015 and divided them into a training set and a test set. In the training set, we processed and screened the predictor variables and addressed the data imbalance by the synthetic minority oversampling technique (SMOTE). Subsequently, we built four machine learning (ML) models to predict SP. After 100 iterations, we selected the best performing model for risk stratification by comparing model discrimination and calibration. Then, we analyzed the relationship between ML risk and SP using scatterplots and logistic regression (LR). Finally, the Shapley’s Additive Explanatory Plot (SHAP) illustrates how each feature level affects the predicted probability of SP.

**Results:**

We ultimately included 1,088 CD older adults, 18.61% of whom reported SP. The optimal model, XGBoost, was selected for prediction and risk stratification. After both univariate (odds ratio [OR]: 12.45, *p* = 4.74 × 10^−10^) and multivariate analyses (OR: 6.98, *p* = 3.96 × 10^−10^), participants with higher ML scores had a higher risk of SP. In sex-specific subanalyses, BMI, height, age, DBP, HDL, etc. were all significant predictors.

**Conclusion:**

This study develops a novel clinically-integrated tool that can be used to easily predict SP in the older adults population with CVD, providing a basis for the development of personalized therapeutic measures.

## 1 Introduction

Cardiovascular disease (CVD) is a group of diseases involving the heart and peripheral vasculature, which is characterized by the interplay of atherosclerosis and myocardial ischemia leading to impaired cardiac function. Statistically, it accounts for 31% of deaths in the global population (1) and up to 40% in China, making it the leading cause of death (2). Previous studies have shown that CVD is associated with irreversible damage and decreased function of multiple organs such as the liver (3) and lungs (4), usually affecting the body’s metabolic, detoxification, and neurological functions. Recent studies have found that chronic inflammatory states and reduced exercise tolerance are associated with CVD; and that the accelerated muscle loss resulting from both is a precursor to the development of sarcopenia (SP), which has become a recognized complication of CVD (5, 6).

Irving Rosenberg first defined SP as muscle atrophy in the older adults (7), and the Asian sarcopenia working group expanded it to include age-related loss of skeletal muscle mass, plus low muscle strength, and/or low physical performance (8). In general, a decrease in skeletal muscle mass usually begins to appear after the age of 40, and the incidence of SP is about 1–33% among people over 50 years old. Among subjects aged 60–70, 5–13% are affected by it, while among older adults aged 80 and above, this proportion can even reach as high as 50%. Studies have shown that SP is widely recognized as a risk factor for CVD and that the overall prevalence of SP in patients with high blood pressure (HBP) and coronary heart disease (CHD) is quite high (9). A systematic evaluation and meta-analysis reported that the prevalence of stroke-related SP was 42% (95% CI 33–52%) and that the prevalence of SP was even higher in the early post-stroke period (10). Research has shown that various characteristics of CVD (such as neuroendocrine disorders, endothelial dysfunction, etc.) affect the balance between protein synthesis and degradation in skeletal muscle, leading to SP (11). These risks can exacerbate the occurrence of related adverse outcomes such as falls, cachexia, and even death in SP patients, and vice versa (12). Therefore, we urgently need to determine methods for early screening of SP in patients with CVD.

Currently, SP diagnosis requires measurement of bone mass. Still, there are some methodological limitations, such as bioimpedance analysis (BIA) which is limited to specific groups of people, and X-ray computed tomography (CT) which exposes the body to radiation and is expensive (13, 14). Currently, there is a paucity of review literature that systematically summarizes and analyzes studies on SP prediction using machine learning (ML) techniques (15). It is worth noting that now with the rapid development of AI-assisted diagnostic technologies, relevant predictive models for this disease have been developed and have high accuracy and sensitivity (13, 16–18). For example, one study used clinical and laboratory metrics data from the West China Health and Aging Trends (WCHAT) study to predict SP using Support Vector Machine (SVM), Random Forest (RF), Extreme Gradient Boosting (XGBoost), and Wide and Deep (W&D) models, and showed that the W&D model had the highest area under the receiver operating curve (AUC) and accuracy (13). Another study using the National Health and Nutrition Examination Survey (NHANES) database in type 2 diabetes developed a novel and practical column chart based on three independent factors: gender, height, and waist circumference, which may be useful to clinicians in predicting the risk of pre-sarcopenia in young people with diabetes mellitus (16). Nevertheless, there is a lack of research on ML’s diagnostic prediction of SP in CVD patients. In addition, although ML has performed well in previous studies, there is limited evidence of its application in Asian populations and interpretable risk prediction models to assist in disease diagnosis. Logistic regression (LR), random forest (RF) (19), support vector machine (SVM) (20), and XGBoost (21) were selected for this study in view of their excellent performance in dealing with high-dimensional and complex data; RF effectively reduces the risk of overfitting, SVM can find the optimal classification hyperplane in high-dimensional space, and XGBoost, with its powerful learning ability and fast training speed, has shown excellent performance in many medical prediction research, and XGBoost has shown excellent performance in many medical prediction studies with its strong learning ability and fast training speed.

In order to address the above limitations, this study develops a diagnostic model for SP in Chinese older adults with the help of an ML approach combined with SHAP in a CVD population. The objectives were to (1) screen the main influencing factors affecting the occurrence of SP in the older adults to provide a basis for early intervention; (2) build a highly interpretable SP diagnostic model to provide a non-invasive, economical, and harmless alternative imaging method for early detection of these patients.

## 2 Methods

### 2.1 Study population

The China Health and Retirement Longitudinal Study (CHARLS), a nationwide longitudinal survey of people >45 years old and their spouses in China, recruited 17,708 participants from June 2011 to March 2012, with multiple follow-ups after that (22). The database used face-to-face computer-assisted personal interviews (CAPI) to examine nearly 10,000 households in 150 counties and 450 villages across 28 provinces in China over an average of 2–3 years, providing information on relevant social, economic, and health status of community residents (22). Ethical approval for all the CHARLS waves was granted from the Institutional Review Board at Peking University. The IRB approval number for the main household survey, including anthropometrics, was IRB00001052-11,015; the IRB approval number for biomarker collection, was IRB00001052-11,014.

**Figure 1:**
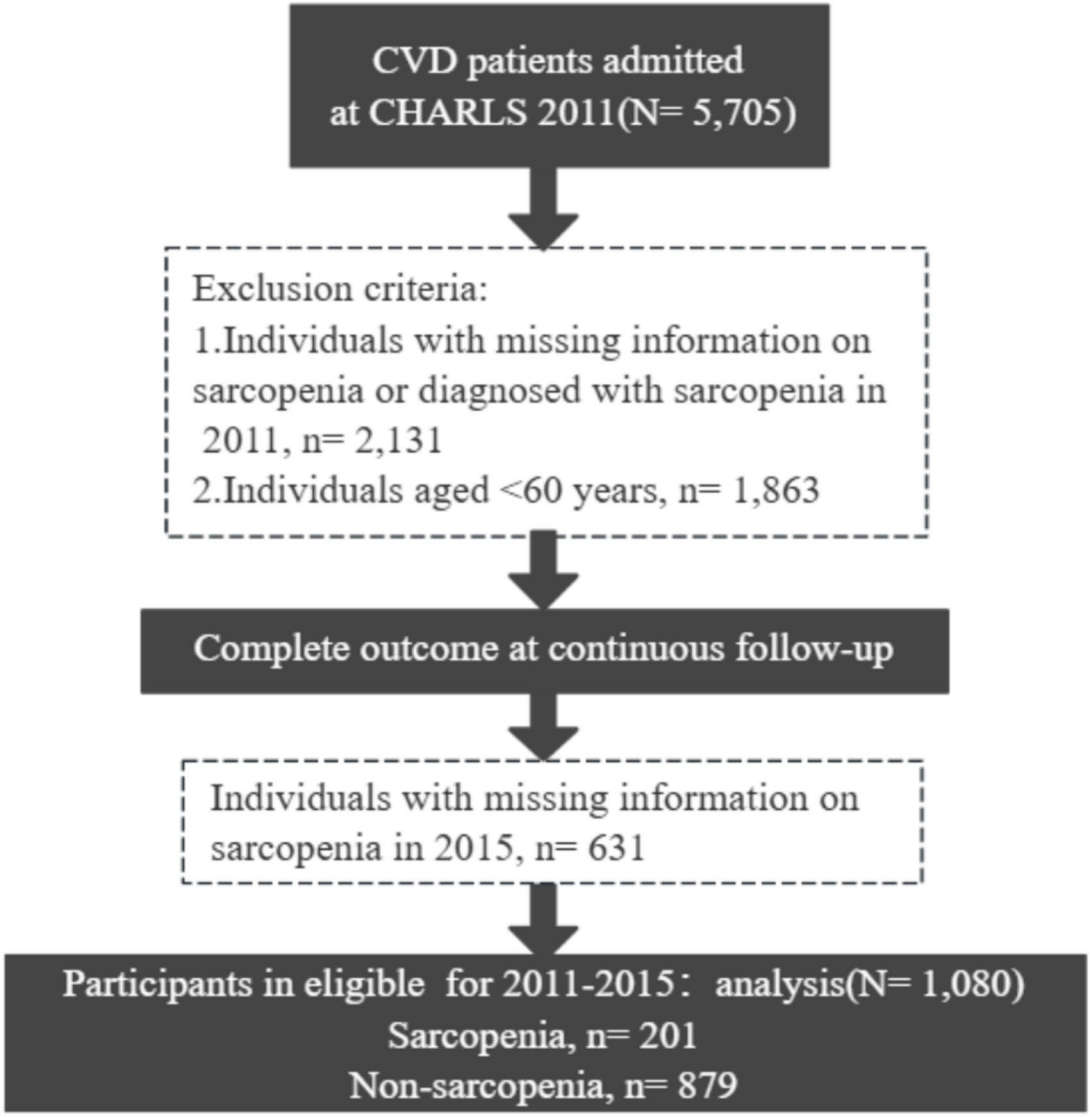
The flowchart for data processing and statistical analysis using the CHARLS in the present work.

Blood tests and physical measurements were performed in the 2011 and 2015 waves, respectively (not available in 2018), so we used the 2011 and 2015 waves to develop a diagnostic model for SP. We excluded participants diagnosed with SP at baseline and included participants aged ≥60 years with a history of CVD (*n* = 1,080) who had blood tests and other health-related data available at baseline (Figure 1).

### 2.2 Ascertainment of outcomes

The diagnostic criteria for SP we defined regarding the latest edition of the Asian Working Group on Muscle Attenuation Syndrome 2019 (AWGS2019) (8), which included three components: low muscle mass (<5.61 kg/m^2^ for females and <7.35 kg/m^2^ for males), muscle strength (<28.0 kg for males and <18.0 kg for females), or low physical performance (gait speed<1 m/s, chair stand test≥12 s) (23, 24).

First, muscle mass was assessed by appendicular skeletal muscle mass (ASM) and height. The equation model of ASM, which was validated on Chinese residents, was in strong agreement with DXA (25, 26):

$\begin{array}{l} \mathrm{ASM}=0.193\times \text{body weight}+0.107\times \text{height}\\ –4.157\times \mathrm{sex}–0.037\times \mathrm{age}–2.631 \end{array}$

Height, weight, and age are given in centimeters, kilograms, and years, respectively. For sex, value 1 represents males and value 2 represents females. Subsequently, we determined the threshold value for low muscle mass based on the lowest 20% of sex for height-adjusted muscle mass (ASM/height^2^) in the study population (26, 27). Xu Wen et al. applied multiple linear regression to develop a height-weight model using DXA-measured ASM as the dependent variable and anthropometric variables as independent variables. The developed regression model was then cross-validated using data from the CV group. The adjusted R^2^ of the equation model was 0.90 and the estimated standard error was 1.63 kg.

Additionally, participants’ muscle strength in their dominant and non-dominant hands was assessed by a Yuejian TM WL-1000 dynamometer (22).

Finally, regarding physical performance, we chose to assess gait speed and chair stand tests. To measure gait speed, participants were asked to walk a 2.5-meter route twice at normal speed and take the average. To measure the chair stand test, participants were asked to stand five times in a row from a 47 cm high chair, keeping their arms crossed in front of their chest, and the time taken (in seconds) was recorded.

### 2.3 Ascertainment of CVD

This study focused on a population of people with CVD and HBP. In previous definitions (28, 29), CVD was identified by assessing two questions, “Have you ever been told by your doctor that you have had a heart attack, angina, CHD, heart failure, or other heart problem?” or “Have you ever been told by a doctor that you have had a stroke?” Study participants also answered the question about HBP, “Have you ever been told by a doctor that you have HBP?”

### 2.4 Feature selection and data preprocessing

To efficiently and comprehensively extract relevant features, we performed feature selection in three categories, including socio-demographic information, physical、exercise, nutritional factors, and clinical factors. Details of the candidate variables measured at baseline for all participants are shown in Supplementary Table 1. The selection of each feature was based on the following factors: (1) the feature had a missingness rate of less than 30% [populated with multiple imputation (30)], We use the mice package in R studio for multiple imputation, where each regression model can be expressed using the following equation:


$$Y_{\text{missing}}=\beta_{0}+\beta_{1}X_{1}+\cdots +\beta_{p}X_{p}+\in$$

where $Y_{\text{missing}}$ missing denotes the missing value of the target variable, $X_{1},\cdots +X_{p}$ denotes the other predictor variables, $\beta_{0},\beta_{1},\cdots +\beta_{p}$ are the regression coefficients and ϵ is the error term. During interpolation, the regression model needs to be appropriately chosen to match the nature of the data (e.g., linear or logistic regression). The error term ϵ is usually assumed to be normally distributed, allowing the interpolation process to reflect the uncertainty in the predictions. Compared to other interpolation methods, it has the following significant advantages: consideration of uncertainty, efficient use of information, applicability to a wide range of analysis methods, and reduction of bias.

(1) The feature was associated with SP or a potential causative factor that turned out to be significant after analyses using logistic regression (LR), and (2) features with low variance and strong correlation (correlation coefficient>0.90) have been remove. The absence of significant linear relationships for the variables was checked using the findLinearCombos function from the Matrix package.

Since different features may have different ranges of values and some ML algorithms are sensitive to the scale of the features, we standardized or normalized the continuous variables and One-Hot the categorical variables.

### 2.5 Derivation and evaluation of prediction models

We predict whether SP occurs or not in four ML models, namely, LR, support vector machine (SVM), random forest (RF), and extreme gradient boosting (XGBoost), which is a scalable tree boosting system that has a wide range of application scenarios in ML. Its loss function does a second-order Taylor expansion of the error part; and also adds a regularization part and parallel selection of each weak classifier, which prevents model overfitting and improves model generalization (31). We randomly divided 1,080 participants into a training set and a test set in a 7:3 ratio. Preprocessing, parameter tuning, and model training were performed on the training set. Considering the imbalance between positive and negative classes, we chose the synthetic minority oversampling technique (SMOTE) (32) for class balancing treatment. The new data are generated by calculating the distance between samples, finding the nearest neighbor samples of a certain minority class of samples, and then by linear interpolation. The specific process is as follows:

Step 1, select a minority class sample: randomly select a sample $x_{i}$ from the minority class sample set.

Step 2, find the nearest neighbor samples: among the minority class samples, find the k nearest neighbor samples of $x_{i}$ by Euclidean distance, and the value of k is set to 5 in this study.

Step 3, randomly select a neighbor sample: from these k nearest neighbor samples, randomly select a sample $x_{\mathrm{nn}}$ .

Step 4, generate a new sample: randomly interpolate between $x_{i}$ and $x_{\mathrm{nn}}$ to generate a new sample according to the following equation. Where λ is a random number between 0 and 1.


$$x_{\mathrm{new}}=x_{i}+\lambda \times (x_{\mathrm{nn}}-x_{i})$$

Step 5, Repeat the above process: repeat steps 1–4, and in our study we set the ratio of the number of diseased to non-diseased samples to 1:1.

**Figure 2:**
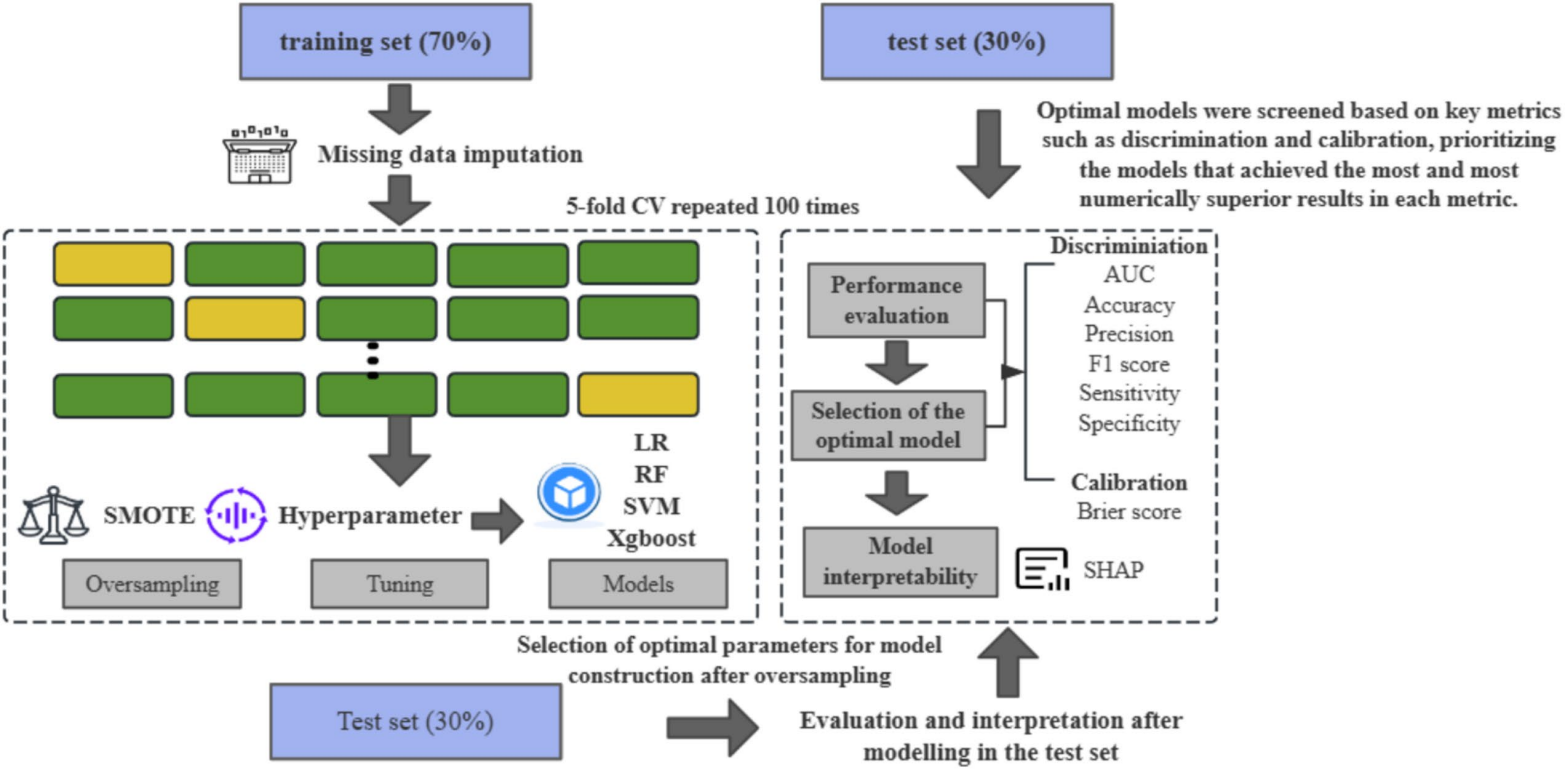
Flow chart of model derivation and test.

After that, this study used a grid search method with a five-fold CV to optimize the hyperparameter combinations, and the specific tuning parameters are detailed in Supplementary Table 2. Finally, we chose the area under the receiver operating characteristic curve (AUC), accuracy, sensitivity, specificity, and F1 scores to assess the model’s discrimination and the Brier scores to assess the model’s calibration. To ensure the robustness of the results and to limit overfitting, we set up 100 different random seeds to repeat the above process and calculate the average performance of these 100 repetitions (33) (Figure 2). In this paper, the TRIPOD process is strictly followed to construct the prediction model (34).

### 2.6 Model interpretation and feature importance

The “black box” problem is considered to be one of the main obstacles to the further development of ML, and we need to increase the interpretability of the results by visualizing the model results both globally and locally. SHapley additive exPlanations (SHAP) (35) assign the contribution of the feature values to the different features. SHAP generates graphical and quantitative interpretations to help interpret the model and make more accurate clinical decisions. Its performance has been validated in previous studies (36). The calculation of SHAP value is based on evaluating all possible combinations of features with the following mathematical formula:


$$\phi_{i}=\sum_{S\subseteq N\setminus \{i\}}\frac{|S|!(|N|-|S|-1)!}{|N|!}(v(S\cup \{i\})-v(S))$$

$\phi_{i}$ is the SHAP value of the features, S is the subset of features (excluding features i ); N is the total set of features; v(S) is the model output of the feature set S , and v(S∪{i}) is the model output after feature set S plus feature i .

Afterward, we calculated the importance of the variables in the optimal model to identify the main predictors of the occurrence of SP in the older adults population with CVD.

### 2.7 Statistical analysis

First, to better understand the data distribution and characteristics, continuous data are presented as means (standard deviations) and were tested using independent samples t-tests or Mann–Whitney U-tests. Categorical variables were presented as frequencies (percentages) and tested using chi-square tests. The highest Youden’s index was used to define the optimal threshold and to differentiate between low-risk and high-risk participants. Scatterplots were then drawn to describe the high and low ML risk distribution. Finally, the relationship between ML risk and 4-year SP was assessed using univariate and multivariate LR (controlling for the top 5 significant predictors). Statistical significance was based on a two-tailed *p* value≤0.05.

All analyses and calculations were performed using R version 4.2.1 (caret, XGBoost, kernlab, randomForest, DMwR, and shapviz packages).

## 3 Results

### 3.1 Patient characteristics of the internal training and testing sets

The baseline characteristics of all participants in this study are shown in Supplementary Table 3. The baseline characteristics of participants after feature screening are shown in Table 1. The data were divided into a training set (70%) and a test set (30%). The prevalence of SP in the older adults population with CVD was 18.61% of the total number of subjects, 18.63% in the training set (141 subjects), and 18.58% in the validation set (60 subjects). The differences between the training and test sets were not statistically significant on the remaining variables such as gender, any weekly contact with children, taking any medications for HBP, CESD scale, visual or hearing problem, complication, BUN, glucose, age, average hours for one night sleeping time during the past month, DBP, height and waist, except for significant differences on HDL (48.53 vs. 45.32, *p* = 0.001) and uric acid (UA) (4.65 vs. 4.86, *p* = 0.018).

**Table 1:** Training and testing set demographics.

Characteristics	Level	Participants, No. (%)			*p* value
		Total (*N* = 1,080)	Train (*N* = 757)	Test (*N* = 323)	
Sarcopenia (%)	No	879 (81.39)	616 (81.37)	263 (81.42)	0.089
	Yes	201 (18.61)	141 (18.63)	60 (18.58)	
Gender (%)	Male	513 (47.50)	346 (45.71)	167 (51.70)	0.082
	Female	567 (52.50)	411 (54.29)	156 (48.30)	
Any weekly contact with children (%)	No	93 (8.61)	63 (8.32)	30 (9.29)	0.690
	Yes	987 (91.39)	694 (91.68)	293 (90.71)	
Takes any medications for HBP (%)	No	407 (37.69)	295 (38.97)	112 (34.67)	0.206
	Yes	673 (62.31)	462 (61.03)	211 (65.33)	
CESD scale (%)	No	630 (58.33)	442 (58.39)	188 (58.20)	0.873
	Yes	450 (41.67)	315 (41.61)	135 (41.80)	
Visual or hearing problem (%)	No	867 (80.28)	603 (79.66)	264 (81.73)	0.483
	Yes	213 (19.72)	154 (20.34)	59 (18.27)	
Complication (%)	No	708 (65.56)	510 (67.37)	198 (61.30)	0.064
	Yes	372 (34.44)	247 (32.63)	125 (38.70)	
BUN (mg/dL) (Mean ± SD)		16.29 (4.42)	16.27 (4.50)	16.32 (4.23)	0.878
HDL (mg/dL) (Mean ± SD)		47.57 (14.03)	48.53 (14.24)	45.32 (13.25)	0.001
Glucose (mg/dL) (Mean ± SD)		117.07 (40.51)	116.47 (39.94)	118.49 (41.86)	0.453
UA (mg/dL) (Mean ± SD)		4.71 (1.28)	4.65 (1.24)	4.86 (1.37)	0.018
Age (y) (Mean ± SD)		66.32 (5.23)	66.26 (5.12)	66.46 (5.47)	0.567
Nst (h, Mean ± SD)		6.19 (1.93)	6.20 (1.95)	6.18 (1.90)	0.848
DBP (mmHg) (Mean ± SD)		78.28 (11.72)	78.37 (11.59)	78.05 (12.06)	0.682
Height (m) (Mean ± SD)		1.57 (0.10)	1.57 (0.08)	1.57 (0.12)	0.555
BMI (kg/m^2^) (Mean ± SD)		26.67 (21.55)	25.82 (4.23)	28.68 (38.85)	0.046
Waist (cm) (Mean ± SD)		91.33 (11.46)	91.45 (11.17)	91.05 (12.13)	0.600

CESD, Center for Epidemiologic Studies Depression Scale; BUN, blood Urea Nitrogen; DBP, diastolic blood pressure; BMI, body mass index; Nst, average hours for one night sleeping time during the past month; UA, uric acid.

### 3.2 ML to predict outcomes

Table 2 shows the prediction performance of the four ML models in the test set. Overall, the new ML models all performed better than the traditional LR model. More specifically, comparing the AUC first, it can be seen from Supplementary Figure S1 that the RF has the highest AUC (all the following values are averages: 0.8769, 95% CI: 0.8367 ~ 0.9171) and the lowest LR (AUC = 0.8459, 95% CI: 0.7922 ~ 0.8996). Next, SVM has the highest precision (0.9681, 95% CI: 0.9429 ~ 0.9932) and specificity (0.9000, 95% CI: 0.8241 ~ 0.9759). However, taken together, the XGBoost had the highest accuracy (0.8050, 95%CI: 0.7575 ~ 0.8467), F1 score (0.8706, 95% CI: 0.8408, 0.9004), sensitivity (0.8061, 95% CI: 0.7583 ~ 0.8539) and Brier score (0.1950, 95% CI: 0.1486 ~ 0.2414) performed best. In addition, its AUC (0.8670, 95% CI: 0.8184 ~ 0.9156) is second only to the RF (Table 2). Therefore, XGBoost was selected for further prediction in this study.

**Table 2:** Scores of each model on the test set.

Indicators	LR	RF	SVM	XGBoost
AUC	0.8459 (0.7922 ~ 0.8996)	**0.8760 (0.8367 ~ 0.9171)**	0.8645 (0.8204 ~ 0.9086)	0.8670 (0.8184 ~ 0.9156)
Accuracy	0.7709 (0.7211 ~ 0.8156)	0.8019 (0.7541 ~ 0.8439)	0.7307 (0.6787 ~ 0.7783)	**0.8050 (0.7575 ~ 0.8467)**
Precision	0.9565 (0.9287 ~ 0.9843)	0.9462 (0.9165 ~ 0.9758)	**0.9681 (0.9429 ~ 0.9932)**	0.9464 (0.9169 ~ 0.9759)
F1 score	0.8426 (0.8096 ~ 0.8755)	0.8683 (0.8382 ~ 0.8984)	0.8071 (0.7707 ~ 0.8435)	**0.8706 (0.8408 ~ 0.9004)**
Sensitivity	0.7529 (0.7007 ~ 0.8050)	0.8023 (0.7541 ~ 0.8504)	0.6920 (0.6362 ~ 0.7478)	**0.8061 (0.7583 ~ 0.8539)**
Specificity	0.8500 (0.7596 ~ 0.9404)	0.8000 (0.6988 ~ 0.9012)	**0.9000 (0.8241 ~ 0.9759)**	0.8000 (0.6988 ~ 0.9012)
Brier score	0.2291 (0.1826 ~ 0.2755)	0.1981 (0.1578 ~ 0.2383)	0.2693 (0.2136 ~ 0.3250)	**0.1950 (0.1486 ~ 0.2414)**

LR, logistic regression; RF, random forest; SVM, Support Vector Machine. Bolded representations perform better in the 4 models.

### 3.3 Categorization of prediction score and risk stratification

**Figure 3:**
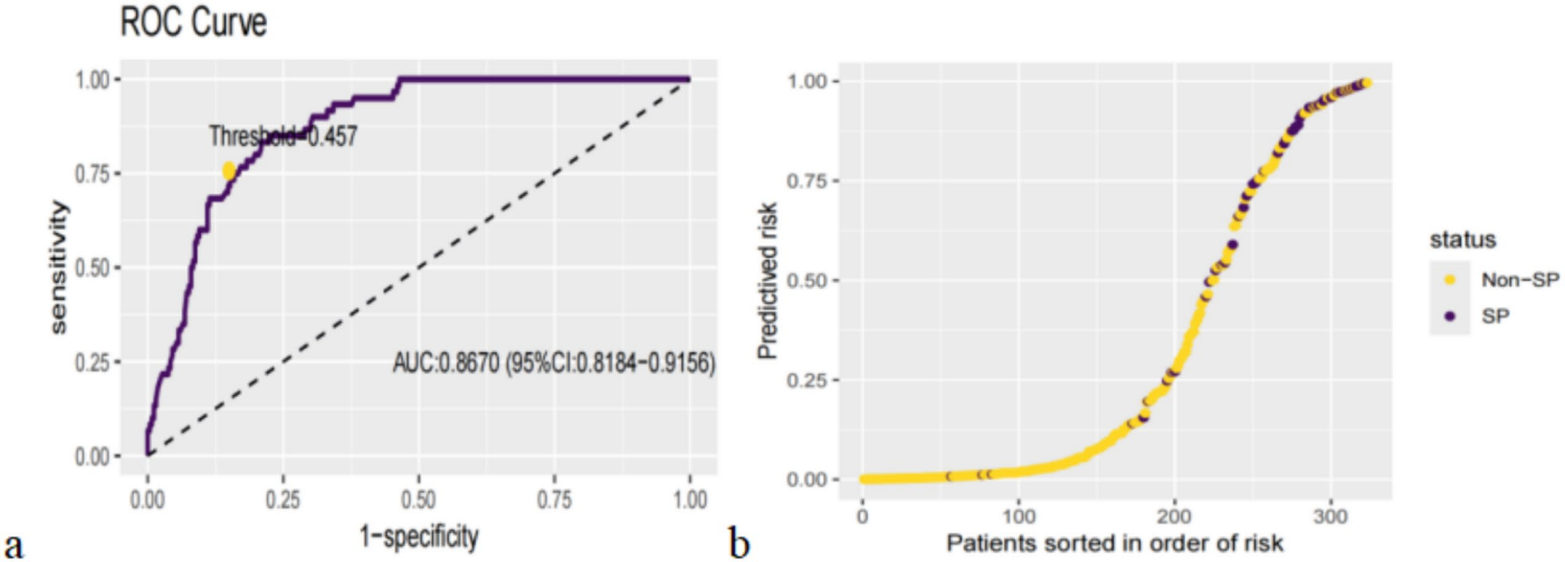
Categorization threshold of prediction score **(a)** and prediction distributions in participants with SP **(b)**.

We categorized patients into high-risk and low-risk groups after establishing the XGBoost in the test set using the maximum Youden’s index as the optimal threshold (0.457, sensitivity = 0.8500, specificity = 0.7985) (Figure 3a). The results of the risk probability scatterplot determined by the optimal model showed a clear aggregation of SP in the older adults population with CVD, further demonstrating the accuracy of the model in stratifying high-risk and low-risk (Figure 3b).

### 3.4 Logistic regression analysis

**Figure 4:**
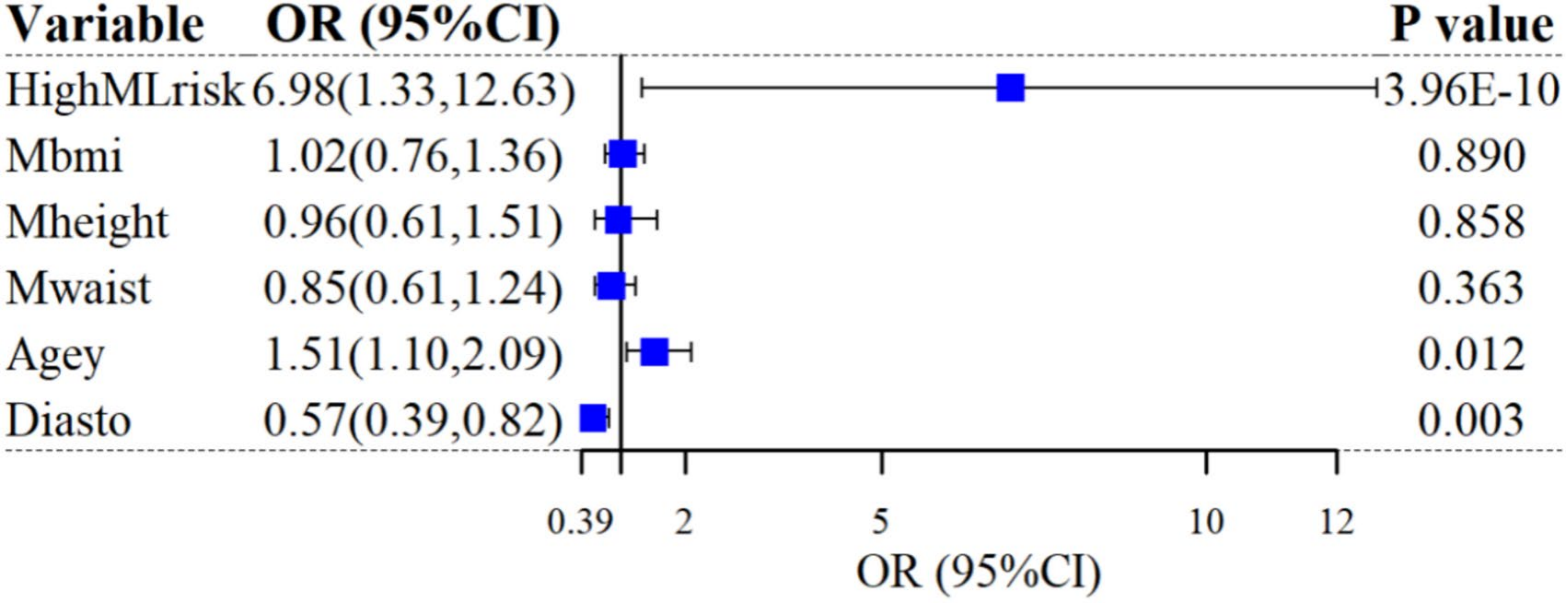
Multivariable logistic regression for SP prediction.

After categorizing participants into low-risk and high-risk based on thresholds in a one-way LR analysis, high ML risk was significantly associated with SP diagnosis (unadjusted odds ratio [OR]: 12.45; 95%CI: 1.87 ~ 23.02; *p =* 4.74 × 10^−10^). After controlling for the five most important predictors (BMI, height, age, waist, and DBP), the correlation remained (adjusted OR: 6.98; 95%CI: 1.33 ~ 12.63; *p =* 3.96 × 10^−10^). The results of the multivariate LR analysis are shown in Figure 4.

### 3.5 Visualization of feature importance and interpretation of personalized predictions

**Figure 5:**
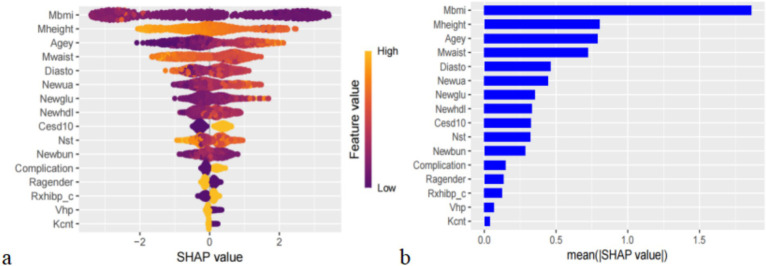
**(a)** Importance of all screened variables according to the mean (|SHA*p* value|), **(b)** the importance of all screened variables using the optimal model.

**Figure 6:**
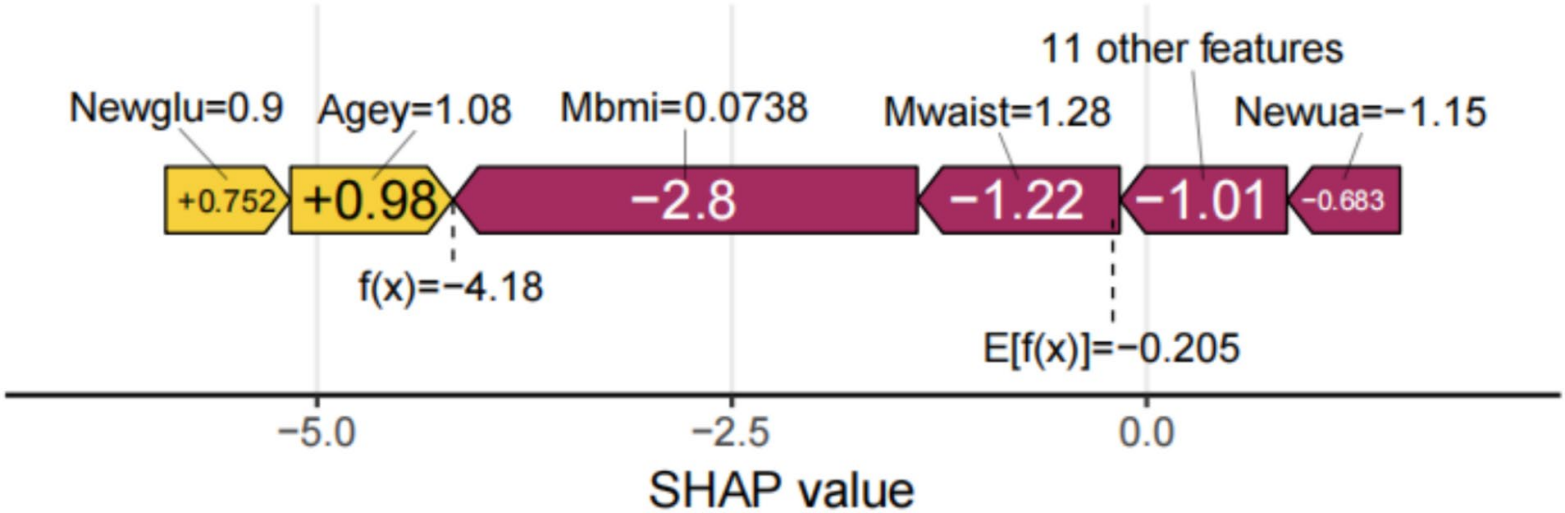
Interpretation of single sample model predictions.

SHAP can globally interpret and visualize the contribution of each feature in the XGBoost model to the prediction. In each feature importance row, all patient attributions to the results are represented by dots of different colors, with purple dots representing a negative correlation between the feature and the result, and yellow dots representing a positive correlation (Figure 5a). Figure 5b shows the importance of all screened variables using the optimal model. *X* and *y*-axis represent a unified index that responds to the influence of a certain feature in the model and variable importance, respectively. Age, UA, glucose, HDL, CESD scale, BUN, complication, taking any medications for HBP, and visual or hearing problems are positively correlated with SP. In contrast, BMI, height, waist, DBP, average hours for one night sleeping time during the past month, gender, and any weekly contact with children were negatively associated with SP (Figure 5a). We also provide an example to illustrate the function of SHAP in locally explaining individual features (Figure 6). Although the specific contribution of each individual may differ, the overall trend remains consistent with what we perceive.

### 3.6 Gender-based analysis

**Figure 7:**
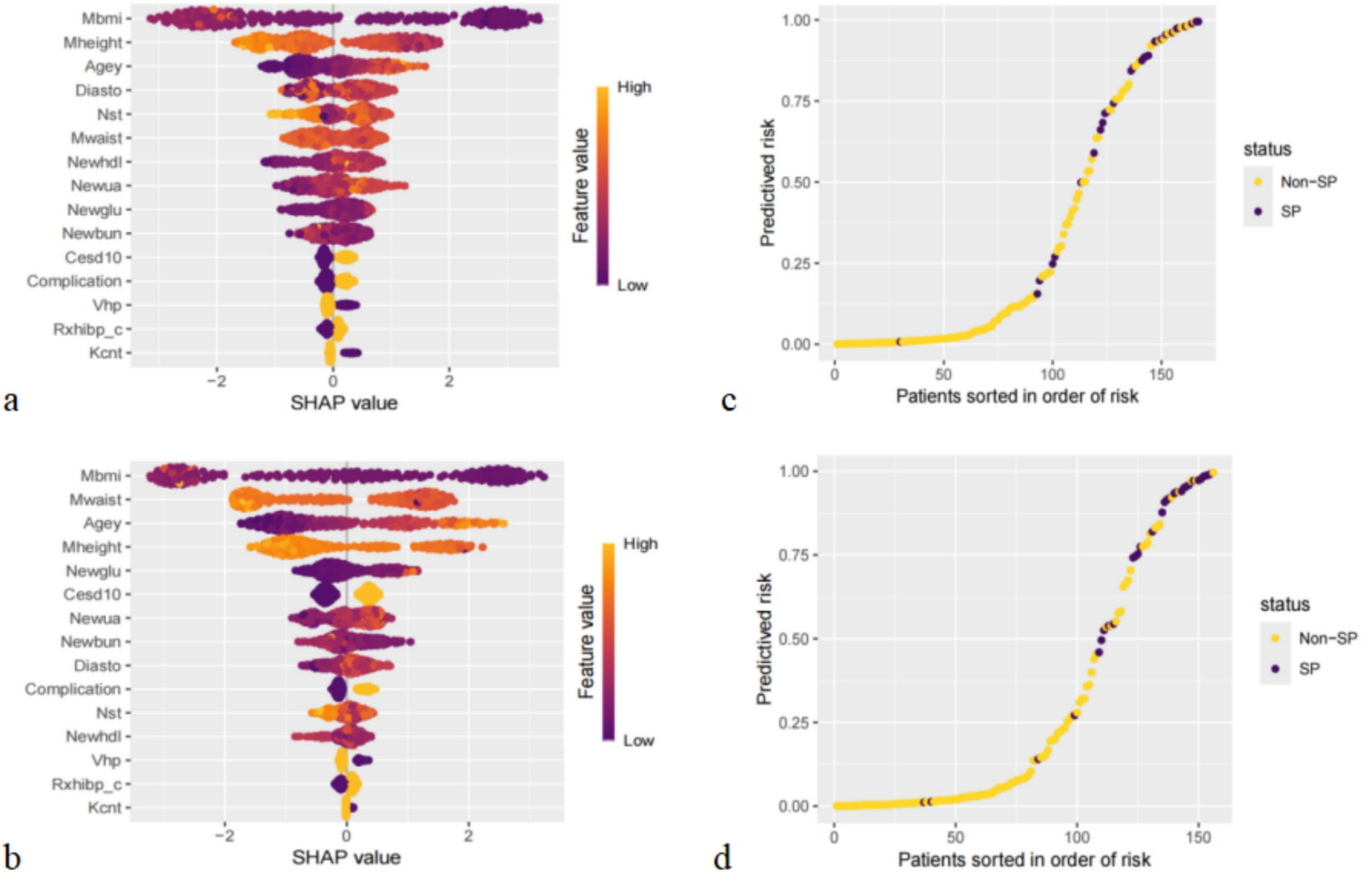
Importance of all screened variables according to the mean (|SHAP value|) for male **(a)** and female **(b)**, and prediction distributions in participants with SP for male **(c)** and female **(d)**.

In the sex-specific subanalysis, we compared the top 10 predictors, average hours for one night sleeping time during the past month and HDL, as significant predictors only in males; they were not among the top 10 predictors in females. Similarly, some factors, such as CESD score and complication, were significant predictors in females only; in males, they were not among the top 10 predictors (Figures 7a,b).

The results of the risk probability scatterplot stratified according to different genders also showed an aggregation of SP in the older adults population with CVD, suggesting that the accuracy of the XGBoost for high-risk and low-risk stratification of different gender groups is also high (Figures 7c,d).

## 4 Discussion

### 4.1 Summary and comparison with existing studies

The occurrence of SP in the real world is still not properly and fully recognized clinically, especially in patients with CVD. Our literature review shows that the number of SP patients in CVD is higher than that in diabetes and respiratory diseases (6). In addition, early stroke is particularly likely to be associated with SP (10), suggesting that early diagnosis and intervention are of great importance in the prevention and treatment of SP in the older adults population with CVD.

In the current study, we developed an interpretable diagnostic prediction model for SP in the older adults population with CVD based on factors such as clinical assessment and socio-demographic information, which may be useful for clinicians to predict the risk of SP in CVD patients. Afterward, performance comparisons were made in terms of both calibration and discrimination of the models, and XGBoost had the best overall performance among the four ML models. The ML risk score generated from this model also indicates high accuracy. In addition, SHAP was used to interpret the features in the model both holistically and locally.

Firstly, regarding the clinical aspects, our study found that lower BMI was a relevant factor for the diagnosis of SP in the older adults population with CVD. Previous studies have shown that lower weight older adults are more likely to have a negative impact on nutritional status due to insufficient protein intake, whereas those with a higher BMI consume sufficient protein which may be protective against SP (37). Also, lower heights also have a higher risk of CVD (38) and the perspective that greater height loss is more likely to be diagnosed with SP has been observed in previous cohort studies (39), which is consistent with our findings. Interestingly, waist is also a correlate of SP because high levels of sex hormones stored within waist fat positively affect skeletal muscle (40), which is consistent with our findings.

The absence of adequate blood supply during diastole due to low DBP may affect the nutritional status of the muscle the lower the DBP the more likely it is to develop into SP (41). A meta-analysis (42) mentioned a significant correlation between HBP and SP in older adults, which is similar to the results of our study, which defined taking HBP-related medications as HBP. Our results also showed a significant association between depressive symptoms (43), visual or hearing impairment (44), and having other comorbidities (renal disease, dyslipidemia, diabetes mellitus, and cancer) (45–48) were predictors of SP, which is a recognized finding in previous studies.

Regarding blood tests, we found that BUN associated with kidney function and glucose associated with diabetes may increase the risk of developing SP. Although the current study has not confirmed that BUN and glucose are associated with skeletal muscle, it is hypothesized that they may further increase the prevalence of SP by affecting renal function and the development of diabetes. Miao Lu et al. (49) demonstrated that UA is a potential risk factor for SP in patients with heart failure with preserved ejection fraction (HFpEF), which is consistent with our findings. Meng Wang et al. (50) noted that for every 1-unit increase in HDL levels, there was a 42% increase in the odds of developing SP, and HDL also showed a positive correlation with SP in the current study.

Regarding socio-demographic information, in addition to gender and age mentioned in previous studies (16, 51), we found that any weekly contact with children in person/phone/emails may be a protective factor. Living alone and social isolation were identified as risk factors for SP by Jiaqing Yang (52). In the Chinese CD, many older adults live alone, and they alleviate their loneliness by talking to their children on the phone, which helps to reduce their risk of SP. After that, regarding physical、exercise, and nutritional aspects, the shorter the average hours for one night sleeping time during the past, the more likely it is to develop SP. Ronaldo D. Piovezan (53) pointed out that with age, the decrease in sleep duration and quality favors protein hydrolysis and increases the risk of insulin resistance, which in turn reduces muscle fibers and strength.

The model constructed in this study brings new ideas for personalized treatment of older adults CVD patients. Through accurate risk stratification, appropriate treatment plans can be formulated according to the different levels of low, medium and high, from low-risk life interventions to high-risk intensive treatment, so as to improve the accuracy of treatment and the efficiency of resource utilization.

### 4.2 Strengths and limitations

Compared with previous studies (54), our work has several advantages. First, considering the possibility of increased sampling error in small, unrepresentative samples, this study used a large Asian-based cohort to increase the accuracy and breadth of the results. Second, the ML model combined with SHAP to analyze the importance and direction of action of each variable in the model helps to identify factors with high impact and improve clinical outcomes through early intervention.

Our study also has limitations. First, in terms of model selection, we did not select deep learning models. In the future, we will try to build deep learning models to predict SP and combine a wider range of data and information for different levels of research. After that, our study lacked external validation from an independent cohort, which may affect the superiority and generalization ability of the model. Finally, in terms of feature selection, we extracted some structured self-reported data and lacked imaging and genetic data. The absence of imaging data, which can visualize muscle morphology and structure (55), and genetic data, which can reflect an individual’s genetic susceptibility (56), may prevent the model from comprehensively capturing the factors affecting the development of SP, thus affecting the accuracy of the prediction. Future studies may consider collecting related data to further improve the prediction model.

## 5 Conclusion

In an older adult CVD population in a Chinese CD, this study demonstrated the feasibility of using advanced ML methods and easily accessible features for effective prediction of SP risk. The ML-based SP risk prediction model can help physicians identify high-risk older adults CVD patients at an early stage, and combined with SHAP values can guide personalized treatment. At the policy level, this model can be used for mass screening to improve the efficiency of resource allocation, and can also promote relevant policies to facilitate the integration of the technology into the clinic.

## Data Availability

The raw data supporting the conclusions of this article will be made available by the authors, without undue reservation.

## Glossary

CVD: Cardiovascular disease
SP: Sarcopenia
HBP: High blood pressure
CHD: Coronary heart disease
BIA: Bioimpedance analysis
CT: Computed tomography
ML: Machine learning
CHARLS: China Health and Retirement Longitudinal Study
CAPI: Computer-assisted personal interviews
ASM: Appendicular skeletal muscle mass
LR: Logistic regression
SVM: Support vector machine
RF: Random forest
XGBoost: Extreme gradient boosting
SMOTE: Synthetic minority oversampling technique
AUC: Area under the receiver operating characteristic curve
SHAP: SHapley additive exPlanations
CESD: Center for Epidemiologic Studies Depression Scale
BUN: Blood Urea Nitrogen
DBP: Diastolic blood pressure
BMI: Body mass index
UA: Uric acid
