# Supplementary material for: Sarcopenia prediction model based on machine learning and SHAP values for community-based older adults with cardiovascular disease in China
Source: Front Public Health. 2025 May 21;13:1527304. doi: 10.3389/fpubh.2025.1527304 (PMC12133505; doi:10.3389/fpubh.2025.1527304)
Supplement: Supplementary file 1 [file Supplementary_file_1.docx]

Supplementary Material

# 1 Supplementary Data

# 2 Supplementary Figures and Tables

# 2.1 Supplementary Tables

Supplementary Table 1 Names and assignments of all candidate variables measured at baseline.

| **Variable** | **Full name/ Features for reintegration** | **Unit /Assignment** |
| --- | --- | --- |
| Sarcopenia | --- | 0: No, 1: Yes |
| Agey | Age | years old |
| Wbc | White Blood Cell in Thousands | 10^9^/L |
| Mcv | Mean erythrocyte volume | (fl) |
| Platelets | Platelets | (L) |
| Newbun | Blood Urea Nitrogen | (mg/dl) |
| Newglu | Glucose | (mg/dl) |
| Newcrea | Creatinine | (mg/dl) |
| Newcho | Total Cholesterol | (mg/dl) |
| Newtg | Triglycerides | (mg/dl) |
| Newhdl | High density lipoprotein Cholesterol | (mg/dl) |
| Newldl | Low density lipoprotein Cholesterol | (mg/dl) |
| Newcrp | Reactive Protein | (mg/l) |
| Newhba1c | Glycated Hemoglobin | (%) |
| Newua | Uric Acid | (mg/dl) |
| Hemat | Hematocrit | (fl) |
| Hemog | Hemoglobin | (g/dl) |
| Cystatin c | Cystatin C | (mg/l) |
| Ragender | Gender | 1:male, 2:female |
| Raeduc_c | Education | 1:unschooled,2:primary/middle school,3:high school and above |
| Mstat | Marital status | 1:married, 2:unmarried |
| Rural | Lives in rural or urban | 0:urban,1:rural |
| Kcnt | Any weekly contact with children in person/phone/ema✔ | 0:no,1:yes |
| Socwk | Participate in social activities | 0:no,1:yes |
| Smokev | Smoke ever | 0:no,1:yes |
| Drinkev | Ever drinks any alcohol before | 0:no,1:yes |
| Nst | Average Hours for One Night Sleeping Time During the Past Month | (h) |
| Nap | How Long Did You Take A Nap During the Past Month | (min) |
| Mwaist | Waist measurement in centimeters | (cm) |
| Mbmi | Measured Body Mass Index | (kg/m2) |
| Rxhibp_c | Takes any meds for high blood pressure | 0:no,1:yes |
| Cesd10 | CESD Score |  |
| Vhp | Visual or hearing problem | 1:yes,2:no |
| Complication | Ever had kidney disease/dyslipidemia/diabetes/cancre | 0:no,1:yes |
| Systo | Average blood pressure measure(systolic) | (mmHg) |
| Diasto | Average blood pressure measure(diastolic) | (mmHg) |
| Pulse | Average pulse measure |  |
| Puff | Maximum lung function peak flow |  |
| Fall | Fallen Down | 1:yes,2:no |
| Cognition | Immediate word recall/delayed word recall/cognition orient/serial 7s/cognition able to draw assign picture/Pain on the Left Side of Your Chest |  |
| Mheight | Measured height in meters | (m) |
| Mweight | Measured weight in kilograms | (kg) |
| Semidone | Completed full 10-second semi-tandem test | 0:no,1:yes |
| Fulldone | Completed 30/60 seconds full-tandem | 0:no,1:yes |
| Adl | Some Diff-Dressing/Diff-Bathing, shower/Diff-Eating/Diff-Get in/out bed/Diff-Using the toilet/Diff-controling urination and defecation | 0:no,1:yes |
| Iadl | Some Diff-Managing money/Diff-Take medications/Diff-Shop for grocery/Diff-Prepare hot meal/Diff-cleaning house/Diff-Use telephone | 0:no,1:yes |

Supplementary Table 2 The optimal hyper-parameters of ML models determined by Grid Search with 5-fold CV.

| **Model** | **Hyperparameters** | **Optimal value** |
| --- | --- | --- |
| XGboost | nrounds = c(100, 200) | 200 |
|  | max_depth = c(1:6) | 4 |
|  | eta = c(0.1:1) | 0.1 |
|  | gamma = c(0:0.5) | 0 |
|  | colsample_bytree = c(0.6:0.9) | 0.6 |
|  | min_child_weight = c(1:9) | 1 |
|  | subsample = c(0.3:0.9) | 0.3 |
|  | objective = "binary:logistic" |  |
|  | eval_metric = "logloss" |  |
| RF | mtry (1:15) | 5 |
| SVM | sigma (0.1,1,10) | 0.100 |
|  | C (0.1,1,10) | 1.000 |

Supplementary Table 3 Training and testing set demographics of all candidate variables.

| **Characteristics** | **Level** | **Participants, No. (%)** | | |  |
| --- | --- | --- | --- | --- | --- |
|  |  | Total  (N =1,080) | Train  (N =757) | Test  (N =323) | *P*-value |
| Ragender (%) | Male | 513 (47.50) | 361 (47.69) | 152 (47.06) | 0.902 |
|  | Female | 567 (52.50) | 396 (52.31) | 171 (52.94) |  |
| Raeduc_c (%) | Unschooled | 556 (51.48) | 400 (52.84) | 156 (48.30) | 0.184 |
|  | Primary/middle school | 456 (42.22) | 315 (41.61) | 141 (43.65) |  |
|  | High school and above | 68 (6.30) | 42 (5.55) | 26 (8.05) |  |
| Mstat (%) | Married | 859 (79.54) | 601 (79.39) | 258 (79.88) | 0.921 |
|  | Unmarried | 221 (20.46) | 156 (20.61) | 65 (20.12) |  |
| Rural (%) | Urban | 459 (42.50) | 312 (41.22) | 147 (45.51) | 0.214 |
|  | Rural | 621 (57.50) | 445 (58.78) | 176 (54.49) |  |
| Kcnt (%) | No | 93 (8.61) | 71 (9.38) | 22 (6.81) | 0.208 |
|  | Yes | 987 (91.39) | 686 (90.62) | 301 (93.19) |  |
| Socwk (%) | No | 551 (51.02) | 398 (52.58) | 153 (47.37) | 0.133 |
|  | Yes | 529 (48.98) | 359 (47.42) | 170 (52.63) |  |
| Smokev (%) | No | 653 (60.46) | 447 (59.05) | 206 (63.78) | 0.165 |
|  | Yes | 427 (39.54) | 310 (40.95) | 117 (36.22) |  |
| Drinkev (%) | No | 665 (61.57) | 471 (62.22) | 194 (60.06) | 0.549 |
|  | Yes | 415 (38.43) | 286 (37.78) | 129 (39.94) |  |
| Rxhibp_c (%) | No | 407 (37.69) | 288 (38.04) | 119 (36.84) | 0.760 |
|  | Yes | 673 (62.31) | 469 (61.96) | 204 (63.16) |  |
| Cesd10 (%) | No | 630 (58.33) | 439 (57.99) | 191 (59.13) | 0.778 |
|  | Yes | 450 (41.67) | 318 (42.01) | 132 (40.87) |  |
| Vhp (%) | Yes | 213 (19.72) | 152 (20.08) | 61 (18.89) | 0.712 |
|  | No | 867 (80.28) | 605 (79.92) | 262 (81.11) |  |
| Complication (%) | No | 708 (65.56) | 498 (65.79) | 210 (65.02) | 0.861 |
|  | Yes | 372 (34.44) | 259 (34.21) | 113 (34.98) |  |
| Fall (%) | Yes | 230 (21.30) | 167 (22.06) | 63 (19.50) | 0.390 |
|  | No | 850 (78.70) | 590 (77.94) | 260 (80.50) |  |
| Fracture (%) | Yes | 14 (1.30) | 11 (1.45) | 3 (0.93) | 0.686 |
|  | No | 1066 (98.70) | 746 (98.55) | 320 (99.07) |  |
| Semidone (%) | No | 22 (2.04) | 17 (2.25) | 5 (1.55) | 0.611 |
|  | Yes | 1058 (97.96) | 740 (97.75) | 318 (98.45) |  |
| Fulldone (%) | No | 324 (30.00) | 217 (28.67) | 107 (33.13) | 0.163 |
|  | Yes | 756 (70.00) | 540 (71.33) | 216 (66.87) |  |
| Adl (%) | No | 15 (1.39) | 11 (1.45) | 4 (1.24) | 0.980 |
|  | Yes | 1065 (98.61) | 746 (98.55) | 319 (98.76) |  |
| Iadl (%) | No | 736 (68.15) | 516 (68.16) | 220 (68.11) | 0.978 |
|  | Yes | 344 (31.85) | 241 (31.84) | 103 (31.89) |  |
| Agey, years old (Mean ±SD) |  | 66.32 (5.23) | 66.23 (5.23) | 66.53 (5.23) | 0.386 |
| Wbc |  | 6.44 (1.96) | 6.46 (1.96) | 6.38 (1.95) | 0.519 |
| Mcv, (fl) (Mean ±SD) |  | 91.25 (7.58) | 91.28 (7.59) | 91.17 (7.57) | 0.814 |
| Platelets, (Mean ±SD) |  | 209.29 (77.08) | 209.37 (77.04) | 209.12 (77.28) | 0.960 |
| Newbun, (mg/dl) (Mean ±SD) |  | 16.29 (4.42) | 16.29 (4.42) | 16.27 (4.41) | 0.939 |
| Newglu, (mg/dl) (Mean ±SD) |  | 117.07 (40.51) | 117.31 (42.69) | 116.51 (34.93) | 0.765 |
| Newcrea, (mg/dl) (Mean ±SD) |  | 0.82 (0.22) | 0.83 (0.23) | 0.81 (0.19) | 0.313 |
| Newcho, (mg/dl) (Mean ±SD) |  | 199.04 (40.10) | 199.19 (40.14) | 198.69 (40.07) | 0.854 |
| Newtg, (mg/dl) (Mean ±SD) |  | 147.66 (110.30) | 147.62 (113.70) | 147.74 (102.08) | 0.987 |
| Newhdl, (mg/dl) (Mean ±SD) |  | 47.57 (14.03) | 47.54 (14.10) | 47.63 (13.88) | 0.930 |
| Newldl, (mg/dl) (Mean ±SD) |  | 122.39 (38.04) | 122.59 (38.52) | 121.94 (36.94) | 0.799 |
| Newcrp, (mg/l) (Mean ±SD) |  | 3.29 (6.27) | 3.37 (6.52) | 3.09 (5.66) | 0.496 |
| Newhba1c, (%) (Mean ±SD) |  | 5.43 (0.88) | 5.42 (0.87) | 5.47 (0.90) | 0.408 |
| Newua, (mg/dl) (Mean ±SD) |  | 4.71 (1.28) | 4.74 (1.32) | 4.66 (1.20) | 0.400 |
| Hemat, (fl) (Mean ±SD) |  | 42.25 (6.12) | 42.44 (6.18) | 41.83 (5.96) | 0.134 |
| Hemog, (g/dl) (Mean ±SD) |  | 14.64 (2.29) | 14.70 (2.37) | 14.52 (2.10) | 0.254 |
| Nst, (h) (Mean ±SD) |  | 6.19 (1.93) | 6.21 (1.91) | 6.15 (1.99) | 0.653 |
| Nap, (min) (Mean ±SD) |  | 38.19 (43.81) | 39.45 (44.07) | 35.24 (43.11) | 0.149 |
| Mwaist, (cm) (Mean ±SD) |  | 91.33 (11.46) | 91.28 (11.44) | 91.44 (11.53) | 0.832 |
| Mbmi, (kg/m2) (Mean ±SD) |  | 26.67 (21.55) | 27.09 (25.61) | 25.70 (3.94) | 0.331 |
| Systo, (mmHg) (Mean ±SD) |  | 141.81 (22.16) | 142.36 (22.60) | 140.51 (21.06) | 0.209 |
| Diasto, (mmHg) (Mean ±SD) |  | 78.28 (11.72) | 78.64 (11.75) | 77.42 (11.64) | 0.118 |
| Pulse, (Mean ±SD) |  | 71.94 (11.07) | 71.84 (11.05) | 72.17 (11.11) | 0.662 |
| Puff, (Mean ±SD) |  | 263.17 (116.92) | 259.47 (114.11) | 271.84 (122.99) | 0.111 |
| Mheight, (m) (Mean ±SD) |  | 1.57 (0.10) | 1.57 (0.10) | 1.58 (0.08) | 0.140 |
| Mweight, (kg) (Mean ±SD) |  | 63.92 (10.24) | 63.84 (10.23) | 64.12 (10.28) | 0.674 |
| Cognition, (Mean ±SD) |  | 15.00 (4.70) | 14.79 (4.62) | 15.51 (4.86) | 0.020 |

# 2 2 Supplementary Figures


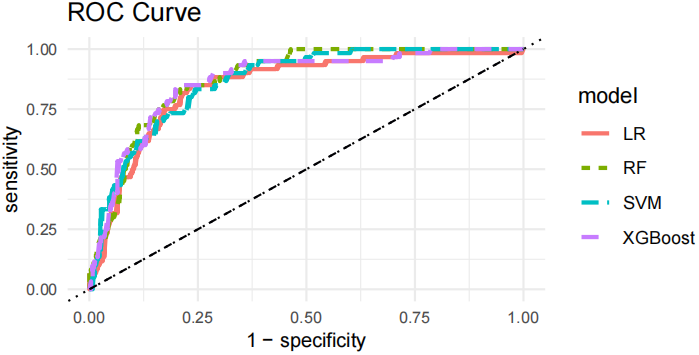


Figure S1 Receiver operating characteristic curves for four models.
